# Alterations in protein conformation assessed by FTIR in EVs from an AD mimicking model

**DOI:** 10.1002/alz.095651

**Published:** 2025-01-09

**Authors:** Margarida Vaz, Tânia Soares Martins, Odete da Cruz e Silva, Alexandra Nunes, Ana Gabriela Henriques

**Affiliations:** ^1^ Neurosciences and Signalling Group, Institute of Biomedicine (iBiMED), Department of Medical Sciences, University of Aveiro, Aveiro Portugal; ^2^ Institute of Biomedicine (iBiMED), Department of Medical Sciences, University of Aveiro, Aveiro Portugal

## Abstract

**Background:**

Aβ peptide is a central player in Alzheimer’s disease (AD) pathogenesis, which once generated rapidly tends to aggregate, from oligomers to fibrils and finally deposits into senile plaques, one of the disease hallmarks.

Extracellular vesicles (EVs) are secreted by all cell types and recognized as key intercellular communication mediators. In AD, it has been reported that EVs can carry Aβ and may potentially accelerate its aggregation, thus contributing to the seeding of the toxic peptide.

Hence, the main goal of this work was to evaluate if Aβ itself can impact the secondary structure of proteins carried by EVs, using Fourier Transform Infrared (FTIR) spectroscopy.

**Method:**

N2a neuroblastoma cells were treated with 10 µM of Aβ_25‐35_ peptide for 48 h, after which EVs were isolated from cell culture medium by ultracentrifugation and then characterized by TEM, NTA and WB. For FTIR analysis, cell suspension samples were also prepared and the FTIR spectra was acquired for N2a and N2a‐derived EVs. The data analysis was focused in the 1700‐1500 cm^−1^ spectral region, which is mostly assigned to protein conformation.

**Result:**

Differences in peak intensities of this spectral region were observed. After Aβ treatment a tendency for an increase in protein aggregation was observed in N2a cells, whereas for EVs a tendency for an increase in protein secondary structures associated with native proteins and oligomers was mostly evident.

**Conclusion:**

These results demonstrated that Aβ can alter the secondary structure of proteins in cells but can also have an impact on the proteins carried by the EVs, affecting protein structures differently. Data reinforces that EVs may be key players in signaling events, involving proteins misfolding, that leads to AD.

This work was funded by Instituto de Biomedicine (iBiMED) under Grant UIDB/04501/2020 and UIDP/04501/2020; MV is supported by the individual PhD grant UI/BD/151354/2021.

